# Progressive Activation of CD127+132− Recent Thymic Emigrants into Terminally Differentiated CD127−132+ T-Cells in HIV-1 Infection

**DOI:** 10.1371/journal.pone.0031148

**Published:** 2012-02-13

**Authors:** Sarah C. Sasson, John J. Zaunders, Nabila Seddiki, Michelle Bailey, Kristin McBride, Kersten K. Koelsch, Kate M. Merlin, Don E. Smith, David A. Cooper, Anthony D. Kelleher

**Affiliations:** 1 The Kirby Institute, The University of New South Wales, Sydney, Australia; 2 HIV Immunopathology Research Laboratory, St Vincent's Hospital Centre for Applied Medical Research, Sydney, Australia; University of Nebraska Medical Center, United States of America

## Abstract

**Aim:**

HIV infection is associated with distortion of T-cell homeostasis and the IL-7/IL7R axis. Progressive infection results in loss of CD127+132− and gains in CD127−132+ CD4+ and CD8+ T-cells. We investigated the correlates of loss of CD127 from the T-cell surface to understand mechanisms underlying this homeostatic dysregulation.

**Methods:**

Peripheral and cord blood mononuclear cells (PBMCs; CBMC) from healthy volunteers and PBMC from patients with HIV infection were studied. CD127+132−, CD127+132+ and CD127−132+ T-cells were phenotyped by activation, differentiation, proliferation and survival markers. Cellular HIV-DNA content and signal-joint T-cell receptor excision circles (sjTRECs) were measured.

**Results:**

CD127+132− T-cells were enriched for naïve cells while CD127−132+ T-cells were enriched for activated/terminally differentiated T-cells in CD4+ and CD8+ subsets in health and HIV infection. HIV was associated with increased proportions of activated/terminally differentiated CD127−132+ T-cells. In contrast to CD127+132− T-cells, CD127−132+ T-cells were Ki-67+Bcl-2^low^ and contained increased levels of HIV-DNA. Naïve CD127+132− T-cells contained a higher proportion of sjTRECs.

**Conclusion:**

The loss of CD127 from the T-cell surface in HIV infection is driven by activation of CD127+132− recent thymic emigrants into CD127−132+ activated/terminally differentiated cells. This process likely results in an irreversible loss of CD127 and permanent distortion of T-cell homeostasis.

## Introduction

The cytokine Interleukin (IL)-7 is non-redundant for T-cell differentiation [1], [2] and plays ongoing roles in T-cell survival through homeostatic [3], [4] and antigen driven proliferation [5]. Circulating IL-7 levels are elevated in lymphopenic conditions [6], [7], [8], suggesting a homeostatic feedback loop and initial studies of therapeutic rIL-7 in oncology and HIV infected patients show promotion of naïve and memory CD4+ and CD8+ T-cell reconstitution [9], [10], [11], [12]. Understanding how HIV infection interacts with the IL-7 receptor subunits CD127 (IL-7Rα) and CD132 (common gamma chain; γc) is therefore relevant to the potential use of rIL-7 as an adjuvant therapy to combination antiretroviral therapy (ART).

HIV infection is associated with a net loss of CD127 from the surface of CD4+ and CD8+ T-cells [13], [14], [15], [16]. This may be secondary to transcriptional down-regulation [10], [17], [18], viral infection [19], [20], antigen stimulation, or IL-7-driven down-regulation promoting endocytosis [21], [22] and/or shedding [23]. The down-regulation of CD127 on T-cells is associated with decreased IL-7-driven proliferation, decreased Bcl-2 expression and cell survival, decreased CD25 expression, and loss of cytotoxic activity [16], [24], [25], [26], [27]. This suggests that IL-7-driven thymopoesis, cellular survival, expansion and generation of central memory T-cells (T(C)M) cells may be inhibited in HIV infection due to lack of an available receptor. Given the development of recombinant IL-7 as an adjuvant therapy, it is important to understand factors that may limit the effectiveness of this cytokine.

Our group recently described novel subsets of T-cells on the basis of IL-7R expression ie CD127+132−, CD127+132+ and CD127−132+. HIV infection was associated with a loss of CD127+132− T-cells and a reciprocal gain in CD127−132+ T-cells in both the CD4+ and CD8+ subsets. These changes were present in primary HIV infection, became more pronounced in chronic HIV infection and were not reversed after 10 months of ART [15].

We sought to understand the phenotypes of CD127+132− and CD127−132+ T-cells and how these differed from the more typical CD127+132+ T-cells. We hypothesised that studying these cells in terms of maturation state, activation, proliferation and survival as well as detecting DNA markers of recent thymic emigration and HIV infection would illuminate why changes in the proportion of these subsets correlate with IL-7 levels and absolute CD4+ T-cell count in HIV infection.

## Materials and Methods

### Subjects

Patients with primary and chronic HIV infection were enrolled in clinical studies at St Vincent's Hospital Sydney. These studies were approved by the institution's Human Research and Ethics Committee (Approval numbers SVH HO2/053 and SVH 96/039) and written informed consent for the use of collected samples was obtained. Peripheral blood mononuclear cells (PBMC) from therapy naïve patients with primary (n = 10) and chronic (n = 10) HIV infection and from healthy volunteers (n = 10) were studied. Cord blood mononuclear cells (CBMC; n = 5) were isolated from healthy infants after uncomplicated births. Patients with primary HIV infection had confirmed recent HIV infection by documented seroconversion illness and incomplete western blot (ie ≤3bands), or negative HIV serology within the preceding 6 months [28]. Patients with chronic HIV infection had been infected with HIV for longer than six months. There were no significant differences in virological or immunological response between patients on different treatment regimes [29]. Contemporaneous clinical data for these patients are shown in Table 1. The primary HIV infection subjects were significantly younger and had a higher baseline CD4+T-cell count compared with the chronic HIV infection subjects as in our previous work [15]. There were no differences is baseline viral load (Table 1).

**Table 1:** Baseline characteristics of patient groups.

	Age (years)	CD4+ T-cell count (cells/µL)	Viral load (copies/ml ×10^3^)
**PHI**	31 (27–33)	675 (418–945)	71400 (20900–690400)
**CHI**	41 (38–45)	281 (117–390)	163750 (32000–296000)
**P value**	<0.05	<0.05	0.82

Median and (interquartile range) are shown. PHI = Primary HIV infection; CHI = Chronic HIV infection.

### Flow-Cytometry

T-cell subsets were identified using multiparameter flow-cytometry in PBMC using the following mAb: CD3-PERCP-Cy5.5, CD4-Alexafluor700 (Pharmingen, San Diego, CA, USA), CD127-Pacific Blue (eBioscience, San Diego CA, USA), CD132-Biotin (Pharmingen, San Diego, CA, USA) with Streptavidin-Quantum Dot-655 (Invitrogen, USA), CD45RO-ECD (Immunotech, Quebec, Canada), mouse anti-human CCR7 (Becton-Dickinson, San Jose CA, USA) with goat anti-mouse-PE (Jackson Immunoresearch, West Grove PA, USA), CD27-PE, CD28-APC, CD25-PECy7, CD31-FITC, CD95-FITC (all Becton-Dickinson, San Jose CA, USA). Extracellular staining was performed as per manufacturers' instructions. Intracellular staining was performed on thawed cryopreserved PBMC as previously described [30].

Flow-cytometry was performed on a dual laser LSR II flow-cytometer (Becton-Dickenson) using FACSDiva v2.2 software. A minimum of 50 000 cells were analysed. Compensation was checked before each experiment and CD127 and CD132 isotype control mAb were also examined. Additional control stains using secondary mAb in the absence of the primary mAb confirmed that there was no non-specific binding.

### Cell-Sorting

Cell-sorting experiments were conducted using a FACS Aria flow-cytometer (Becton-Dickenson). 15–30×10^6^ PBMC from healthy volunteers or patients with primary HIV infection were thawed and stained as described above. Unstained cells and those stained with a single fluorochromes were used to set compensation and positive and negative gates prior to each cell-sort. Compensation was confirmed using cells stained with all CD3-PECRP-Cy 5.5, CD4-Alexaflour700, CD127 Pacific Blue and CD132-PE. PBMC for sorting were stained with mAb to CD3, CD4 CD127 and CD132 as described above. The sorted populations collected were CD3+4+127+132−, CD3+4+127+132+ and CD3+4+127−132+. CD3+4+45RO−62L+ naïve and CD3+4+45RO+62L− memory T-cells and unsorted PBMC were also collected as assay controls. The purity of sorted populations was generally >95%.

### Signal-joint T-cell receptor α excision circle (sjTREC) detection by real-time PCR

DNA was purified from T-cell populations using the Qia kit (Qiagen, Hilden, Germany) according to manufacturer's instructions. Signal-joint (Sj) TRECs were measured by real-time quantitative PCR as previously described [31]. Briefly, each PCR reaction was conducted in a 20 µL mixture containing 12.5 µL of 2× Taqman Mastermix, 1.25 µL each of forward primer, reverse primer and probe (containing a quencher and reporter dye sybr green) and 3.75 µL of water. The sequences of the primers and probe used previously [31] are: forward primer 5′-CCATGCTGACACCTCTGGTT-3′, reverse primer 5′-TCGTGAGAACGGTGAATGAAG-3′ and the probe 5′-CACGGTGATGCATAGGCACCTGC-3′. To nomalise for the amount of DNA the Cα constant region of the TCR was also amplified using the following: forward primer 5′-CCTGATCCTCCTGTCCCACAG-3′, reverse primer 5′-GGATTTAGAGTCTCTCAGCTGGTACA-3′, and the probe 5′-ATCCAGAACCCTGACCCTGACCCTGCCG-3′. The PCR conditions were: 50°C for 2 min; 95°C for 10 min; 50 cycles of amplification (95°C for 15 s; 60°C for 1 min). All samples were run in duplicate on a Rotogene PCR machine (Corbett, Australia) and all runs included a non-template control. For each sample run in duplicate the Ct-value, defined as the minimal number of cycles necessary to exceed threshold values was measured and averaged and then a ratio of sjTREC: Cα was recorded.

### HIV-DNA assay

Real-time quantitative PCR was used to measure the total *gag* HIV-1 DNA as previously described [32]. Briefly, a 25 µl reaction containing 12.5 µL of iQ supermix mastermix (Bio-Rad, USA) and a final concentration of 800 nM of forward and reverse primers and 200 nM of probe and 5 µl of target was used. The sequences were as follows: forward primer 5′-AGTGGGGGGACATCAAGCAGCC-3′, reverse primer 5′-TACTAGTAGTTCCTGCTATGTCACTTCC-3′ and probe 5′-FAM-AT[C]A[A]T[G]AGGAA[G]CT[G]C-TAMRA-3′. HIV-1 DNA was normalised using the ABI TaqMan β-actin detection reagents kit (Applied Biosystems, USA) in 25 µl with a final concentration of 120 nM of forward and reverse primers and 180 nM of a FAM labelled probe and 5 µl of template. The PCR conditions were: 3 minutes at 95°C for 1 cycle, followed by 40 cycles of 95°C for 15 s and 60°C for 1 minute. All samples were run in duplicate and all runs included no template controls and were quantified using standard curves of pNL4-3 plasmid for HIV-1 DNA and a non-infected PBMC buffy coat standard curve for β-actin DNA on a Bio-Rad iQ5 thermocycler (Bio-Rad, USA).

### Statistics

Differences between groups were determined using the unpaired non-parametric Mann-Whitney rank (for two groups), or Kruskal-Wallis rank test (for three groups). All were performed using StatView Data Analysis and Presentation V5.0 software (Abacus concepts, Berkley, USA). A p value<0.05 was considered statistically significant and were not corrected for multiple comparisons.

## Results

### Enrichment of naïve T-cells in the CD4+127+132− and T(E)M and TTD T-cells in the CD4+127−132+ T-cell compartment

We confirmed, in a separate cohort, our previous finding that HIV infection was associated with a net loss of CD127+132− T-cells and a reciprocal gain in CD127−132+ T-cells using a 9-colour panel (data not shown). CD127+132− and CD127−132+ T-cells were plotted on CD45RO vs CCR7 histograms to determine the naïve (CD45RO−CCR7+), effector memory (T(E)M; CD45RO+CCR7−), central memory (T(C)M; CD45RO+CCR7+) or terminally differentiated (TTD; CD45RO−CCR7−) phenotype.

**Figure 1:**
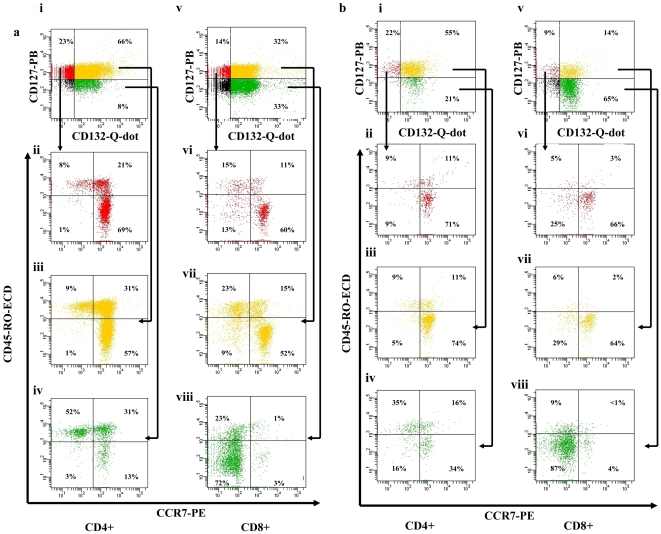
Phenotype of CD127+132−, CD127+132+ and CD127−132+ T-cells based on extracellular expression of CCR7 and CD45RO in a) health and b) chronic HIV infection (representative raw data). Data collected from thawed cryopreserved peripheral blood mononuclear cells (PBMCS) are shown. Cells were initially gated on CD3 vs side-scatter histograms to isolate lymphocytes before being plotted on CD3 vs CD4 histogram to separate CD4+ (ii–iv) and CD8+ (v–viii) lymphocytes (via negative CD4 gating). Data representative of 20 subjects are shown.

In healthy volunteers CD4+127+132− T-cells contained the greatest proportion of naïve cells (median:71%) compared with CD127+132+ (61%) or CD127−132+ T-cells (45%; p<0.01). This remained true in primary (CD127+132−: 60% naïve, CD127+132+: 54%, CD127−132+:30%; p<0.001) and in chronic HIV infection (CD127+132−: 53% naïve, CD127+132+: 46%, CD127−132+:20%; p<0.05; Figure 1a)ii and b)ii).

In healthy volunteers CD4+127−132+ T-cells contained the greatest proportion of T(E)M cells (median:30%) compared with CD127+132− (18%) or CD127+132+ (25%; p<0.05). In primary (CD127+132−: 18% T(E)M, CD127+132+: 25%, CD127−132+:27%; p = 0.13) and chronic HIV infection T(E)M cells were more evenly spread (CD127+132−: 18% T(E)M, CD127+132+: 25%, CD127−132+:35%; p = 0.06; Figure 1a)iii and b)iii).

The CD4+127−132+ T-cells also contained the greatest proportion of T(C)M T-cells (median:22%) compared with CD127+132− (10%) and CD127+132+(18%), although this did not reach significance (p = 0.07). A similar distribution was noted in primary (CD127+132−: 15% T(C)M, CD127+132+:17%, CD127−132+:28%; p = 0.05) and chronic HIV infection (CD127+132−: 22% T(C)M, CD127+132+: 20%, CD127−132+:34%; p = 0.32; Figure 1a)iv and b)iv).

CD127−132+ T-cells contained the greatest proportion of TTD (median:2%) as compared with CD127+132− or CD127+132+ T-cells (both median:1%; p<0.05). This difference became more pronounced in primary (CD127+132−: 4% TTD, CD127+132+: 3%, CD127−132+:14%; p<0.05) and chronic HIV infection (CD127+132−: 7% TTD, CD127+132+: 3%, CD127−132+:9%; p<0.05; Figure 1a)iv and b)iv).

### Enrichment of naïve T-cells in the CD8+127+132− compartment and TTD T-cells in the CD8+127−132+ T-cell compartment

In the CD8+ compartment, CD127+132− T-cells contained the greatest proportion of naïve cells (median:62%) compared with CD127+132+ (50%) or CD127−132+ T-cells (10%; p<0.0001) in healthy volunteers. This trend was maintained in primary (CD127+132−: 40% naive, CD127+132+: 43%, CD127−132+:6%; p<0.0001) and chronic HIV infection (CD127+132−: 27% naive, CD127+132+: 27%, CD127−132+:3%; p<0.001; Figure 1a)vi and b)vi).

In the CD8+ T-cell compartment T(E)M T-cells were spread between CD127+132−(median:11%), CD127+132+ (16%) and CD127−132+ (13%;p = 0.13)T-cells. The T(E)M T-cells remained spread across these subsets in primary (CD127+132−: 20% T(E)M, CD127+132+: 23%, CD127−132+:13%; p = 0.46) and chronic HIV infection (CD127+132−: 15% T(E)M,CD127+132+: 23%, CD127−132+:8%; p = 0.17). In healthy volunteers there was a suggestion that CD127−132+ contained the greatest proportion of T(C)M cells (median:33%) compared with CD127+132− (14%) or CD127+132+ (13%) T-cells, but this did not approach significance (p = 0.77). This distribution was similar in primary HIV infection (CD127+132−: 13% T(C)M, CD127+132+: 12%, CD127−132+:25%; p = 0.85), and in chronic HIV infection where it did reach significance (CD127+132−: 15% T(C)M, CD127+132+: 19%, CD127−132+:25%; p<0.05; Figure 1a)vii and b)vii).

CD8+127−132+ T-cells contained the greatest proportion of TTD (median:44%) as compared with CD127+132− (7%) and CD127+132+ T-cells (9%; p<0.001). This remained true in primary HIV infection (CD127+132−: 19% TTD, CD127+132+: 18%, CD127−132+:39%; p<0.05), and was more marked in chronic HIV infection (CD127+132−: 27% TTD, CD127+132+: 23%, CD127−132+:59%; p<0.01; Figure 1a)viii and b)viii).

### Impact of HIV infection on the phenotype of CD4+ T-cell subsets

**Figure 2:**
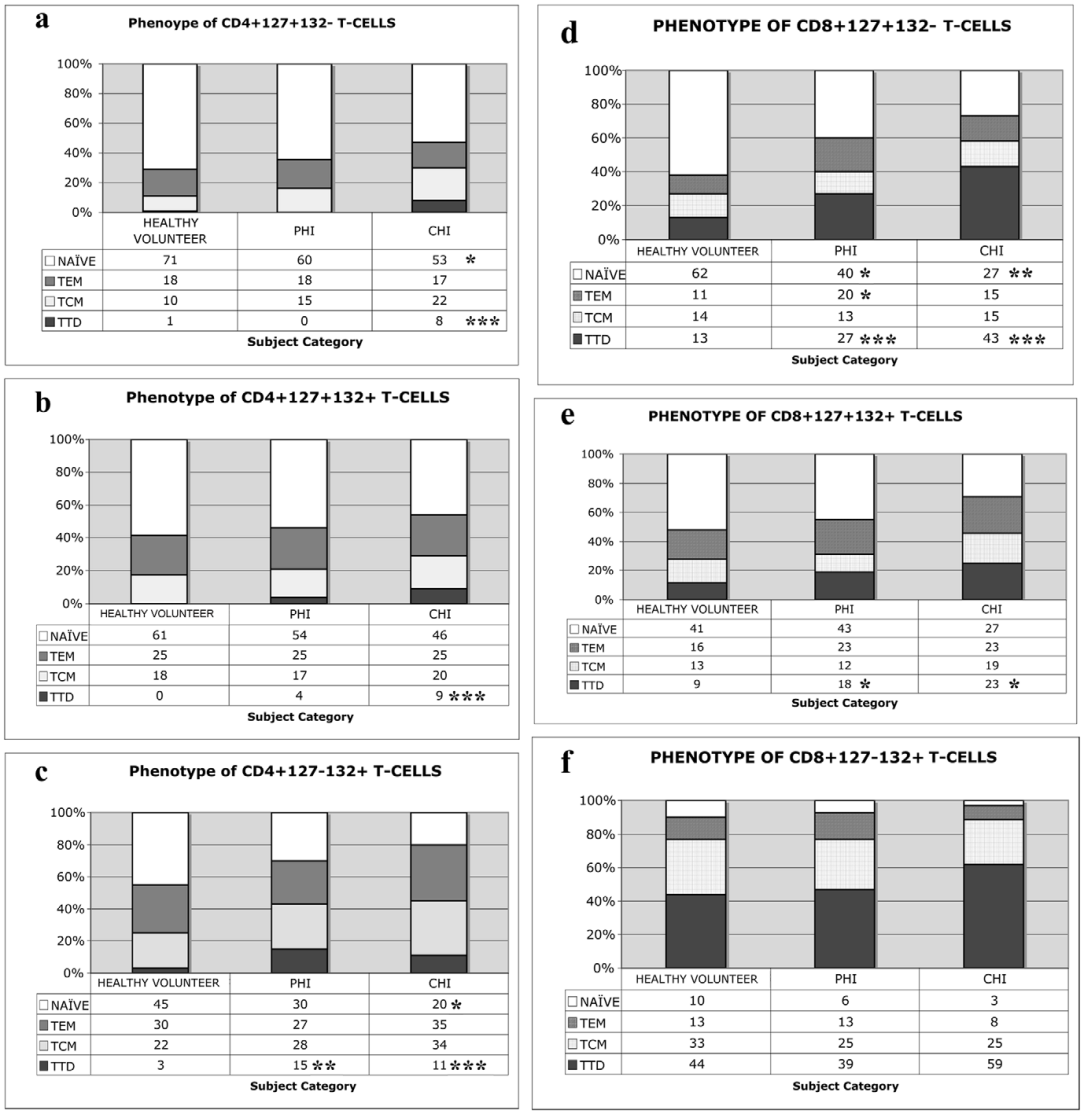
Phenotype of CD127+132−, CD127+132+ and CD127−132+based on extra-cellular expression of CCR7 and CD45RO in health and HIV infection at baseline (Group Data). HIV-associated changes in T-cell populations based on CCR7, CD45RO and IL-7R component expression are shown in a–c) CD4+ and d–f) CD8+ T-cell compartments. PHI = primary HIV infection; CHI = chronic HIV infection. *p<0.05; **p<0.01 and ***p<0.001 as compared with healthy volunteers and determined by the non-parametric Mann-Whitney rank test.

The majority of CD4+127+132− T-cells in healthy volunteers have a naïve phenotype. HIV infection was associated with a reduction in the proportion of naïve cells and an increase in the proportion of TTD in this subset (Figure 2a). The CD4+127+132+ compartment was relatively unaffected by HIV infection, but there was an increase in TTD seen in chronic HIV infection (Figure 2b). HIV infection impacts significantly on the CD4+127−132+ compartment with a loss of cells with a naïve phenotype and a gain in the proportion of TTD cells (Figure 2c). T-regulatory cells (T-reg; CD25+ CD127^low^) are another subset of CD127−132+ CD4+ T-cells [33], [34]. This subset remains constant in healthy volunteers (median: 3% of CD4+127−132+ T-cells), primary (2%) and chronic (3%; p = 0.76) HIV infection. Therefore, the expansion of CD4+127−132+ T-cells is not due to an expansion of T-reg cells.

### Impact of HIV infection on the phenotype of CD8+T-cell subsets

The majority of CD8+127+132− T-cells in healthy volunteers have a naïve phenotype. HIV infection results in a reduction in the proportion of naïve cells and a gain in the proportion of TTD within this subset. Primary HIV infection is also associated with a transient increased in T(E)M cells in this compartment (Figure 2d). The distribution within the CD8+127+132+ T-cell compartment is relatively unaffected by HIV infection, but there is a slight increase in TTD seen in chronic HIV infection (Figure 2e). In the CD8+127−132+ T-cell compartment there is a trend towards loss of cells with a naïve phenotype and a gain in the proportion of TTD cells although this does not reach statistical significance (Figure 2f).

### Increased expression of extra-cellular markers of activation and terminal differentiation on CD127−132+ T-cells

**Figure 3:**
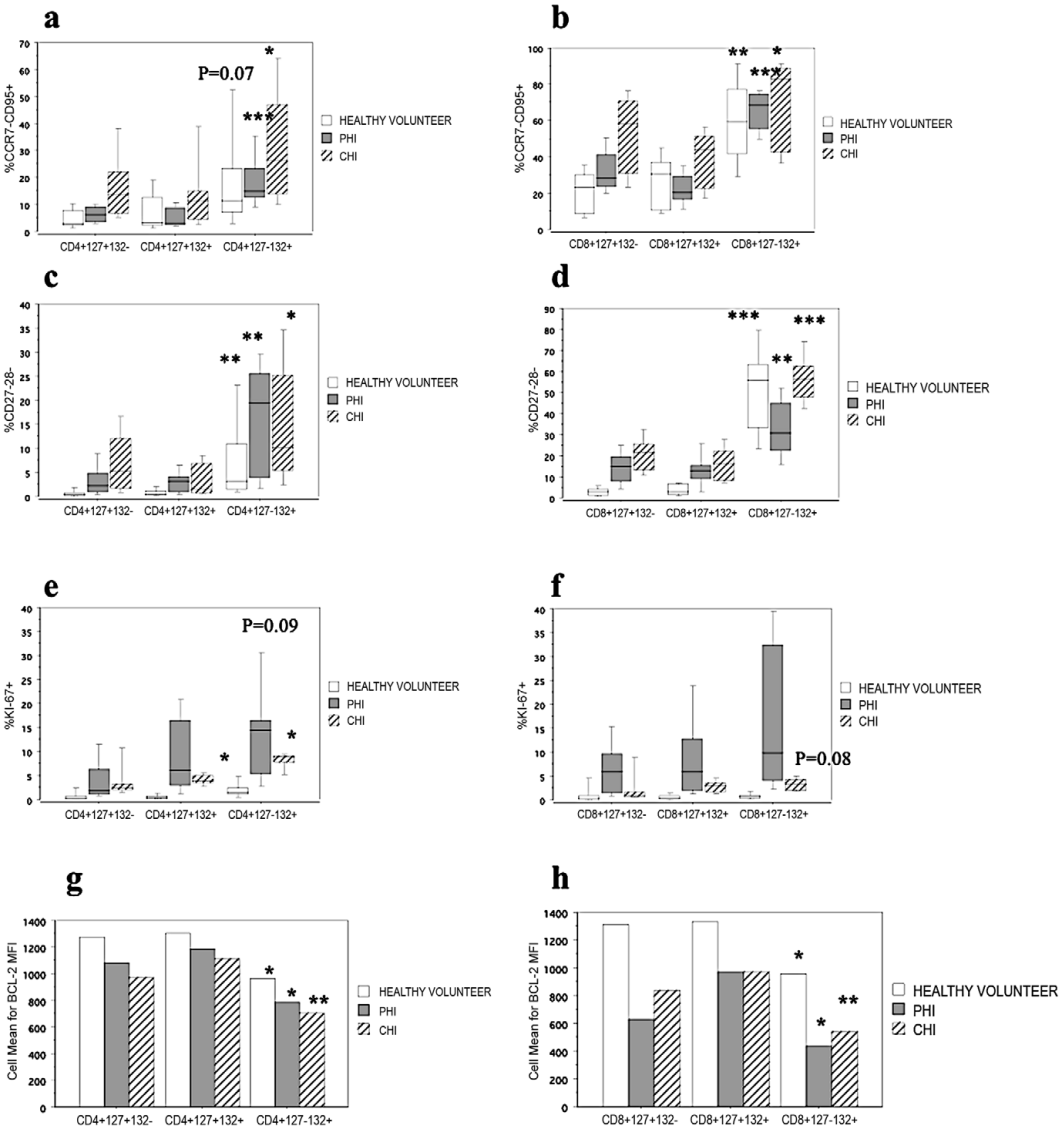
Activation phenotype of CD127+132−, CD127+132+ and CD127−132+based on extra-and intracellular markers in health and HIV infection at baseline. HIV-associated changes in T-cell populations based on a–b) CCR7 and CD95 c–d) CD27 and CD28 e–f) Ki-67 g–h)Bcl-2 and IL-7R component expression are shown in a,c,e,g) for CD4+ and b,d,f,h) for CD8+ T-cell compartments. PHI = primary HIV infection; CHI = chronic HIV infection. *p<0.05; **p<0.01 and ***p<0.001 within each subject category by the non-parametric Kruskal-Wallis test.

CD127−132+ T-cells had a significantly greater proportion of CCR7−CD95+ activated T-cells as compared with CD127+132− and CD127+132+ counterparts. This was true in health, primary and chronic HIV infection, in both CD4+ (Figure 3a) and CD8+ (Figure 3b) T-cell compartments. Similarly, CD127−132+ T-cells also had a significantly greater proportion of CD27−28− terminally differentiated T-cells as compared with CD127+132− and CD127+132+ counterparts. This was true in health, primary and chronic HIV infection, in both CD4+ (Figure 3c) and CD8+ (Figure 3d) T-cell compartments.

### Increased markers of cellular proliferation and decreased markers of survival in CD127−132+ T-cells

CD127−132+ T-cells had a significantly greater proportion cells that had recently proliferated (ie Ki-67+), as compared with CD127+132− and CD127+132+ counterparts. This was true in the CD4+ (Figure 3f) but not CD8+ T-cell (Figure 3g) compartments. In the CD4+ T-cell compartment CD127−132+T-cells has the greatest proportion of Ki-67+ cells in health and chronic HIV infection, however this did not reach significance in primary HIV infection, where there was a greater amount of proliferation in all subsets. Interestingly in the CD8+ T-cell compartment there was global proliferation of T-cells from all subsets during primary HIV infection consistent with a robust acute response to infection. In contrast there was minimal proliferation in chronic HIV infection, perhaps suggesting anergised cells.

Additionally, CD127−132+ T-cells had a significantly lower proportion of cells expressing the anti-apoptotic protein Bcl-2, as compared with CD127+132− and CD127+132+ counterparts. This was true in health, primary and chronic HIV infection, in both CD4+ (Figure 3g) and CD8+ (Figure 3h) T-cell compartments.

### Increased levels of HIV-1 DNA in CD4+127−132+ T-cells

As the CD4+127−132+ T-cells that were expanded in HIV infection were largely T(E)M or TTD cells, we examined whether these activated cells were more likely to be infected with HIV. In four patients with primary HIV infection we measured the amount of HIV-DNA in CD127+132−, CD127+132+ and CD127−132+ CD4+ T-cells. We found that CD4+127+132− T-cells had a relatively low amount of HIV-DNA (median:488 copies/500 ng DNA) which was increased greater than 4-fold in CD4+127+132+ T-cells (2228 copies/500 ng DNA) and in CD4+127−132+ T-cells (2444 copies/500 ng DNA; p = 0.23).

### Higher concentration of sjTRECs in CD4+127+132− T-cells compared with other subsets

**Figure 4:**
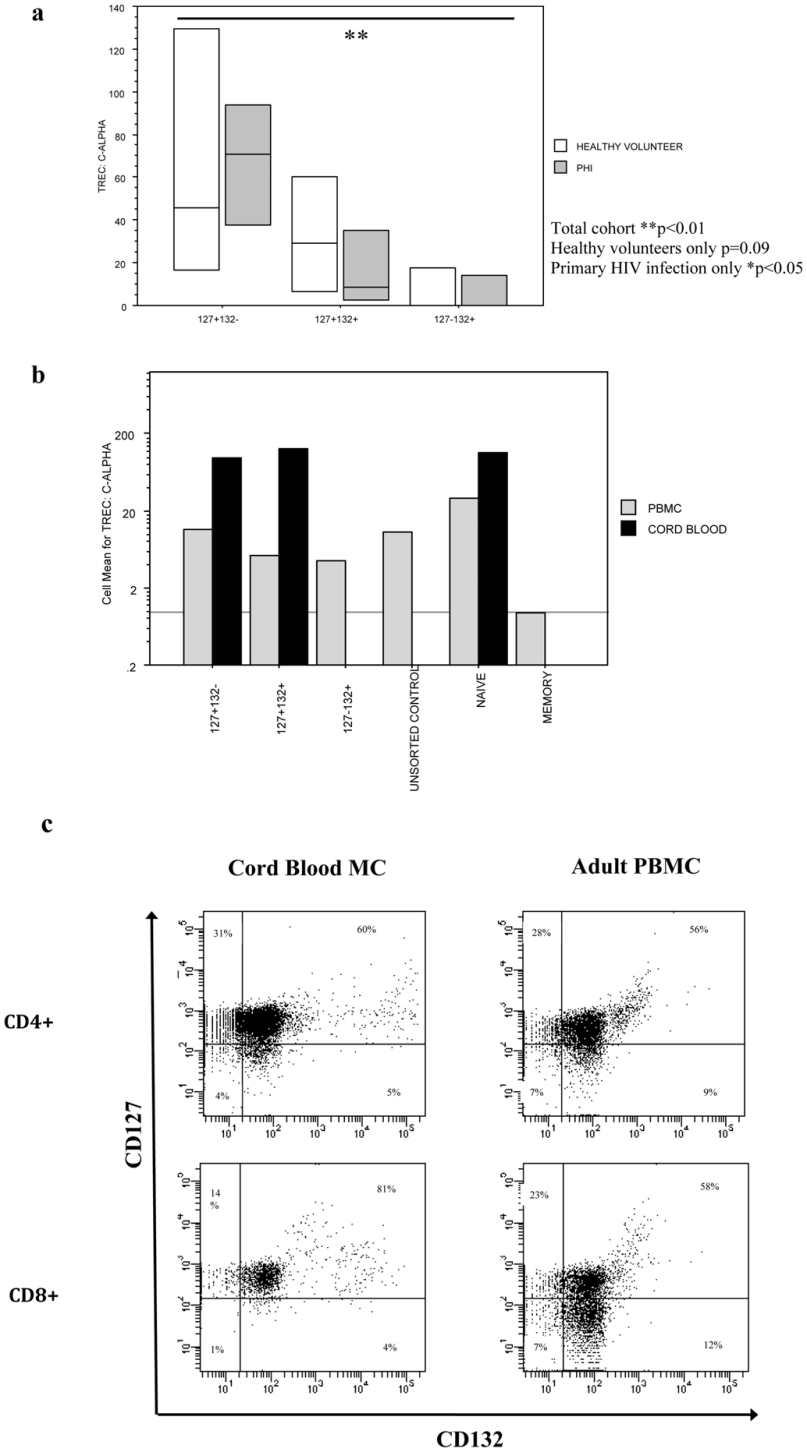
CD4+127+132− T-cells are enriched in Cord Blood mononuclear cells and contain a high proportion of sjTRECS compared with CD127+132+ and CD127−132+ T-cells. a–b) CD4+127+132− T-cells are enriched for sjTRECS in healthy adult volunteers and patients with primary HIV infection. Cryopreserved PBMCs from healthy volunteers (N = 4) or patients with primary HIV infection (PHI; N = 4) were sorted into CD4+127+132−, CD4+127+132+ or CD4+127−132+ populations. DNA was then extracted and the TREC: C-α ratio was determined by real-time PCR as shown in the box-plots below. b) Additional proof of principle experiments confirm the higher concentration of TRECs in naïve CD4+ T-cells as compared with memory T-cells as shown in bar charts below. *p<0.05; **p<0.01 as measured by the Kruskal Wallis non-parametric test. Cord Blood mononuclear cells had a high concentration of TRECS that were not enriched in the CD127+132− subset again as shown in bar charts. c) Cord blood mononuclear cells have a trend towards a greater proportion of CD127+132− CD4+ T-cells and a smaller proportion of CD127−132+ CD4+ T-cells as compared with adult PBMCs. Data representative of 10 subjects.

To determine whether CD127+132− T-cells were in fact recent thymic emigrants we sorted CD4+ T-cells from healthy volunteers and patients with primary HIV infection into CD127+132−, CD127+132+ and CD127−132+ subsets and then measured sjTRECS as a ratio compared to the C-α housekeeping gene. Overall there was a significantly higher concentration of sjTRECs in the CD127+132− subset compared with CD127+132+ or CD127−132+ CD4+ T-cells (p<0.01; Figure 4b). When the healthy volunteers were analysed alone, this approached significance (p = 0.09) and was statistically significant in the primary HIV infection cohort (p<0.05).

In further proof-of-principle experiments we show that the level of sjTRECs in unsorted population of T-cells was intermediate as compared with our sjTREC rich CD127+132− and sjTREC depleted CD127−132+ T-cells (Figure 4b). Additionally we showed that T-cells with a naïve phenoytpe (CD45RO−62L+) had far higher concentration of sjTRECs than those with memory phenotype (CD45RO+62L−) and that CD4+ cord blood T-cells have a higher proportion of sjTRECs than adult PBMC counterparts, both as expected (Figure 4b).

### Relative lack of CD127−132+ T-cells in cord blood mononuclear cells

Finally we hypothesised that the proportion of both CD127+132− and CD127−132+ T-cells would be significantly altered in cord blood mononuclear cells as compared with adult PBMC due to the relative lack of antigen exposure and T-cell activation *in utero*. There was a higher proportion of CD127+132− CD4+ (median = 27%) and CD8+ (24%) T-cells in cord blood as compared with PBMC (21% CD4+ p = 0.17; 18% CD8+ p = 0.34), however this did not reach significance. There were also significantly less CD127−132+ CD4+ (6%) and CD8+ (5%) T-cells in cord blood compared with adult PBMC (10% CD4 p<0.01; 24% CD8+ ; p<0.01).

## Discussion

HIV infection is associated with down-regulation of CD127 from the surface of CD4+ and CD8+ T-cells [13], [14], [15], [16]. It is unknown whether this is the result of viral infection, antigen stimulation or ongoing ligand stimulation by elevated levels of IL-7. However this down-regulation of CD127 is associated with marked alterations in T-cell homeostasis, particularly decreased Bcl-2 induction and cell survival and loss of CTL activity [16], [24], [25], [26], [27]. Therefore determining what drives CD127 down-regulation may have important implications for understanding T-cell homeostasis, as well as on the application of therapeutic IL-7.

Our group recently showed that HIV infection was associated in a progressive loss in the proportion of CD127+132− T-cells and a reciprocal gain in the CD127−132+ T-cells which correlated to increased circulating IL-7 levels and decreased absolute CD4+ T-cell counts and did not reverse following ART [15].

Here we report that CD127+132− T-cells are enriched for CCR7+45RO− naïve cells in CD4+ and CD8+ T-cell compartments in both health and HIV infection. Conversely, CD127−132+ T-cells were most enriched for CCR7−CD45RO−CD27−CD28− terminally differentiated memory T-cells in CD4+ and CD8+ compartments in healthy and HIV infected hosts.

In the CD4+ T-cell subset CD127−132+ T-cells were associated with a greater proportion of Ki-67+ proliferating cells in healthy volunteers and chronic HIV infected patients, however in primary HIV infection there was global proliferation of cells across all subsets. In the CD8+ T-cell compartment there was little proliferation in healthy volunteers, marked global proliferation in primary HIV infection (likely representing a primary immune response), and a relative lack of proliferation of the CD127−132+ T-cells in chronic HIV infection which may represent a state of “exhausted” T-cells as seen in other chronic viral infections [35], [36], [37]. The anti-apoptotic protein Bcl-2 was lower in the CD127−132+ subset in health and HIV infection in both CD4+ and CD8+ T-cell subsets, indicating these cells are short-lived. Additionally our data suggests a relative segregation of HIV-DNA in CD127−132+ and CD127+132+ CD4+ T-cells compared with CD127+132− T-cells during primary HIV infection, although this requires confirmation in larger numbers.

Our data suggests that CD127+132− are a subset of naive cells and we questioned whether the CD127+132− T-cells represented recent thymic emigrants. Indeed sjTREC analysis showed that CD127+132− had the highest proportion of sjTRECs compared with CD127+132+ and CD127−132+ CD4+T-cells this was true in health and primary HIV infection.

In summary this work suggests that T-cells exit the thymus as CD127+132− T-cells that are largely Ki-67− and Bcl-2^high^ and lacking expression of activation markers. As these cells undergo antigen driven activation they are more likely to have a CD127+132+ phenotype, and the most activated/terminally differentiated cells are CD127−132+ that are largely Ki-67+Bcl-2^low^ and enriched for CD95+ and CD27−28− terminally differentiated cells. We tested this hypothesis by measuring T-cell populations in cord blood mononuclear cells (which would have been exposed to minimal antigens) and comparing them to adult PBMC. Cord blood showed a trend towards expansion of naïve CD127+132− CD4+ and CD8+ T-cells and also a significant decrease in the proportion of activated CD127−132+ CD4+ and CD8+ T-cells.

**Figure 5:**
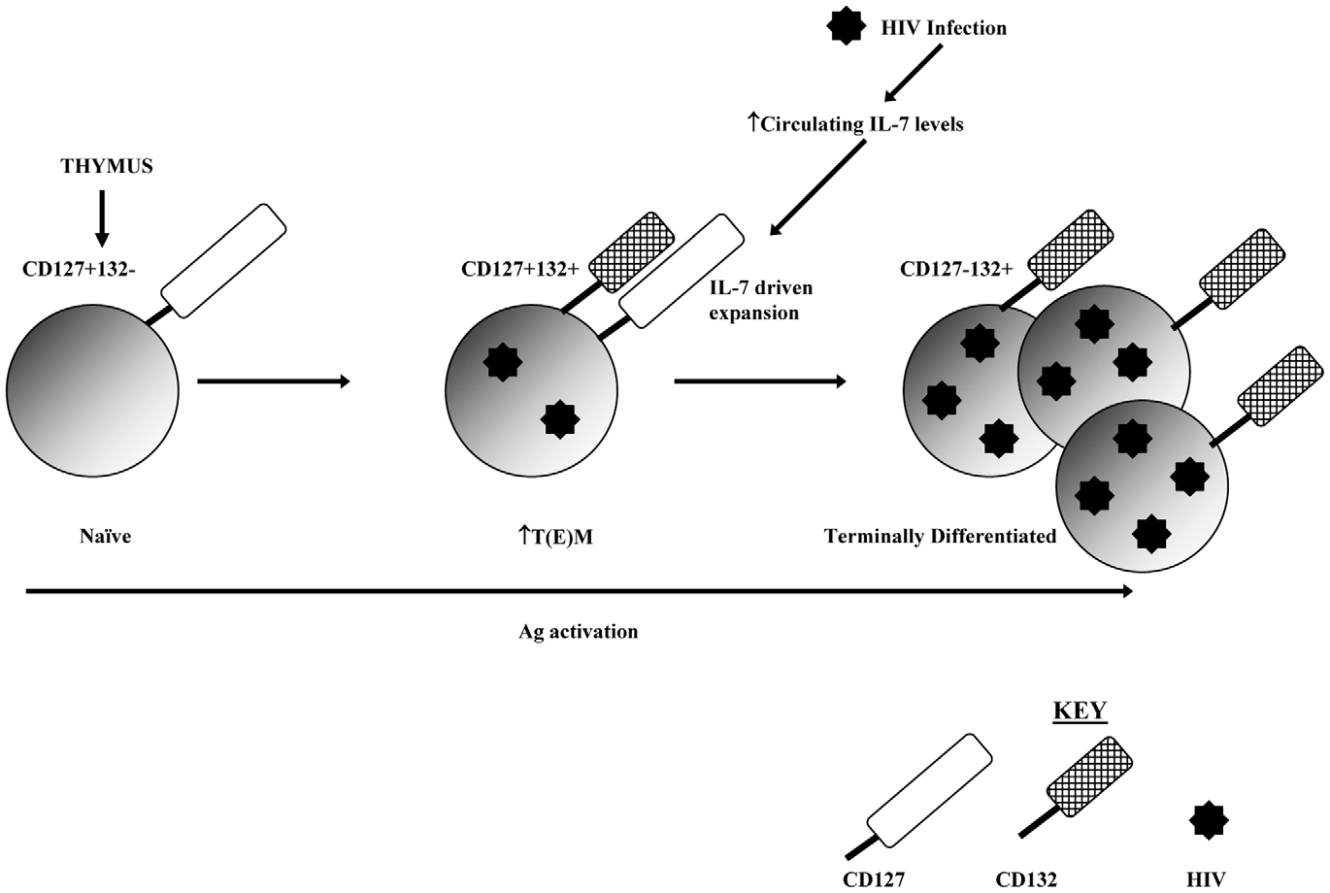
Proposed model for loss of CD127+132− and gain of CD127−132+ T-cells in HIV infection. The data presented here suggest that T-cells exit the thymus expressing CD127 but not CD132. As these recent thymic emigrants mature through antigen (Ag) activation they co-express CD127 and CD132. The continuous antigenic activation and high circulating IL-7 levels associated with HIV infection results in ongoing activation and expansion of these CD127+132+ cells which progress to terminally differentiated CD127−132+ T-cell. As HIV preferentially infects activated cells these terminally differentiated CD127−132+ T-cells contain the greatest amount of viral DNA.

HIV infection impacts on this model in two ways: firstly there is a net loss of naïve cells and a net gain of TTD, concurrently there is a loss of CD127+132− T-cells and a gain in CD127−132+ T-cells. HIV infection therefore expands the proportion of CD127−132+ activated/terminally differentiated T-cells which occur to a smaller degree in the healthy host. Additionally our data suggests HIV infection is preferentially segregated within this subset possibly due to their highly activated state during a primary immune response. This model is summarised in Figure 5.

Given the phenotype of CD127−132+ T-cells are highly activated/terminally differentiated, it is more likely that repeat antigen activation from HIV infection is the main driving force behind the net loss of CD127 from the T-cell surface of these cells. While IL-7 ligand binding alone has also been shown to down-regulate CD127 from the cell surface, this does not cause classical activation of the T-cell and instead cells undergo homeostatic driven proliferation which includes T-cells dividing and retaining a naïve phenotype [38], [39]. However it is also true that IL-7 can synergise with antigen stimulation to cause expansion of T(E)M cells [5], and in this instance IL-7 may synergise with HIV antigen to drive expansion of newly activated CD127- T-cells. Certainly this may explain why plasma IL-7 levels correlate positively with the proportion of CD4+127−132+ and negatively with CD4+127+132− T-cells [15].

In conclusion this work has delineated that the net loss of CD127 expression in HIV is driven at least in part by the activation of sjTREC rich CD127+ naïve cells into more activated phenotypes which may be preferentially infected by HIV. Our initial work showed the populations depleted by HIV infection do not recover following 10 months of ART suggesting the transition from CD127+ naïve cells to CD127− terminally differentiated/ activated and infected T-cells is irreversible, at least in the short to mid-term. These data may be relevant to studies of therapeutic IL-7, which may be less useful in advanced disease where the pool of CD127+ T-cells is more greatly diminished.
